# Relationship between bruxism and mandibular bone modifications based on medical imaging: a scoping review

**DOI:** 10.1186/s12903-023-03209-2

**Published:** 2023-07-14

**Authors:** Estelle Casazza, Benoit Ballester, Blanche Siaud, Camille Philip-Alliez, Anne Raskin

**Affiliations:** 1 Aix Marseille University, APHM, CNRS, EFS, ADES, 27 boulevard Jean Moulin – 13 385, Marseille, France; 2 Aix Marseille University, APHM, INSERM, IRD, SESSTIM, ISSPAM, Marseille, France; 3 Aix Marseille University, Marseille, France; 4 Aix Marseille University, APHM, University Gustave Eiffel, LBA, Marseille, France

**Keywords:** Bone density, Bruxism, Panoramic radiography, Cone-beam computed tomography, Mandible

## Abstract

**Objectives:**

This scoping review aimed to assess the current state of knowledge regarding the relationship between bruxism and changes in density or volume of mandibular bone, based on medical imaging.

**Methods:**

Literature review was conducted following the PRISMA-ScR protocol. PubMed, Web of Science and Cochrane library databases were searched for peer-reviewed articles by two blinded reviewers. Studies based on the evaluation of mandibular bone density and/or bone volume with imaging examination in adult patients were examined. The selected articles were summarized in PICOS tables and assessed for methodological quality.

**Results:**

Nine articles were included, according to the inclusion criteria. They showed that bruxer patients had more bony exostoses of the mandibular angle, smaller condyles, and morphological changes for cancellous and cortical mandibular bone compared to non-bruxer patients.

**Conclusion:**

Bruxism seems to induce morphological and anatomical changes in the different regions of the mandibular bone (condyles, mandibular angle, mandible body). Given the heterogeneity of the included studies, these results should be interpreted with caution. Further studies are needed to support these results, in particular via the analysis of three-dimensional imaging to overcome the limitations of panoramic radiograph.

## Background

The manducatory apparatus, because of the great diversity of its anatomical components, can be the site of several types of symptomatology affecting the patient's quality of life: sequelae of facial trauma [1], temporomandibular disorders, with a complex multifactorial aetiology [2, 3]. It is also concerned by bruxism.

Bruxism is defined as “a repetitive masticatory muscle activity characterized as forcefully maintaining a certain mandibular position and thrusting as forcefully moving the mandible in a forward or lateral direction—both activities without the necessary presence of tooth contact” [4]. It is a widespread phenomenon that may affect around 20% of the adult population and 33% of children [5, 6]. This high prevalence has attracted the interest of the scientific community for several decades, with the aim of improving understanding and management [7].

In spite of the negative image conveyed by its first scientific descriptions at the beginning of the XXth century (it was then qualified as "bruxomania", revealing a psychological disorder [8]), then considered only a parafunction deleterious to the dental system and prosthetic restorations, bruxism has for several years been dissociated from this description to be considered as belonging to a biological continuum [9]. Indeed, it should no longer be considered a disorder but rather an orofacial behaviour that can represent a risk and/or a protective factor with certain clinical consequences. Thus, depending on the patient, bruxism can be considered by the practitioner as:

- A function in the context of habitual, "commonplace" bruxism,
- A parafunction, in the context of active, "frequent" bruxism,
- A pathogenic function in individuals with a fragile dental structure associated with severe, "excessive" bruxism.

When the mandibular movements produced during bruxism episodes by contraction of the mandibular elevator muscles generate inter-arch dental contacts, the force developed by the bruxer can be up to three times higher than during the functional activity of the manducatory apparatus [10–13]. These forces of greater intensity, duration and frequency, will be transmitted to the teeth as well as to the supporting tissues that constitute the periodontium, including alveolar bone, and will have different types of repercussions [14]. Thus, these loads can induce an architectural modification of mandibular bone tissue. This phenomenon was described by Wolff in 1892. Wolff's law states that bone is able to adapt its external cortical and trabecular structure in accordance with the loads to which it is subjected [15]. Thus, in humans, bone variability depends on two characteristics: an innate element, mediated by genetic inheritance, and an acquired element, mediated by behaviour, which includes bruxism [16]. This bone remodelling can be observed through changes in various characteristics of bone tissue, principally variations in volume or density [17].

Moreover, several methods exist for the diagnosis of bruxism, with varying degrees of reliability (self-administered questionnaire, clinical examination, medical examinations (electromyography or polysomnography) [4]: all require a living patient. Only one method is available for the study of bone tissue characteristics: medical imaging.

The objective of this scoping review was to assess the current state of knowledge regarding the relationship between bruxism and changes in density or volume of mandibular bone, based on medical imaging.

## Materials and methods

This review was carried out according to the Preferred Reporting Items for Systematic Reviews and Meta-Analyses extension for Scoping Reviews (PRISMA-ScR) protocol [18, 19].

### Research question and eligibility criteria

The PICOS tool, which stands for Population, Intervention, Comparison, Outcome, and Study type, is detailed in Table 1. It was used to pose the research question, "Is there a difference in mandibular bone density or volume in adult patients diagnosed as bruxers that can be objectified by imaging?".

**Table 1:** Development of the research question based on PICOS

PICOS Question	
Population	Adult patients diagnosed as bruxers (by medical questionnaire and/or clinical examination and/or polysomnography)
Intervention	Analysis of imaging examination (2D or 3D): panoramic radiograph or cone beam computed tomography
Comparison	Density or volume of mandibular bone, without regard to the region of interest considered
Outcome	Evaluation of a difference in the volume or density of mandibular bone
Study type	Randomized or non-randomized clinical trials and observational studies (cross-sectional and longitudinal, retrospective or prospective)

The inclusion criteria were:

- Adult patients,
- Patients diagnosed as bruxers or non-bruxers, without regard to the method used to diagnose bruxism,
- Patients who had undergone medical imaging of the mandible,
- Articles written in English,
- Studies approved by an ethics committee.

The exclusion criteria were:

- Studies including patients with progressive or degenerative pathologies of the mandibular bone (cancer, osteoporosis, etc.), fractures of the mandible or a history of oral radiotherapy,
- Single clinical case study, case report, literature review,
- In vitro or animal model study,
- Documents other than a scientific article (thesis, book…).

There were no restrictions on the year of publication.

### Data collection

The search of scientific articles was conducted in three online databases, PubMed, Web of Science Cochrane library, up to February 05, 2023 by two blinded operators. Studies were excluded on the basis of titles and abstracts, and upon reading the full article. All phases were independently assessed by two evaluators (EC and BS), and in case of doubt or disagreement, a consensus between the two evaluators was sought.

The search procedure used the keywords "bruxism" and "bruxer" together with keywords related to medical imaging examinations and others related to changes in the characteristics of the mandibular bone.

Search equation for PubMed:

("bruxism"[TIAB] OR "bruxer*"[TIAB]).

AND ("radio*" OR "panoramic*" OR "scanner*" OR "CBCT" OR "cone-beam" OR "cone beam" OR "Radiography"[Mesh] OR "Radiography, Dental"[Mesh] OR "Radiography, Panoramic"[Mesh] OR "Cone-Beam Computed Tomography"[Mesh] OR "Tomography, X-Ray Computed"[Mesh]).

AND ("bone density" OR "bone height" OR "bone dimension*" OR "surface area" OR "cortical index" OR ("fractal" AND ("analysis" OR "dimension*")) OR "radiomorphometric indice*" OR "morphological characteristic*" OR "craniomorphological characteristic*").

Search equation for Web of Science:

TS = ("bruxism" OR "bruxer*").

AND ALL = ("radio*" OR "panoramic*" OR "scanner*" OR "CBCT" OR "cone beam").

AND ALL = ("bone density" OR "bone height" OR "bone dimension*" OR "surface area" OR "cortical index" OR ("fractal" AND ("analysis" OR "dimension*")) OR "radiomorphometric indice*" OR "morphological characteristic*" OR "craniomorphological characteristic*").

Search equation for Cochrane library: ("bruxism" OR "bruxer*") in Title Abstract Keyword AND ("radio*" OR "panoramic*" OR "scanner*" OR "CBCT" or "cone beam") in All Text AND ("bone density" OR "bone height" OR "bone dimention*" OR "surface area" OR "cortical index" OR ("fractal" AND ("analysis" OR "dimention*")) OR "radiomorphometric indice*" OR "morphological characteristic*" OR "craniomorphological characteristic*") in All Text.

### Qualitative analysis of results

A qualitative analysis of protocols is offered in this scoping review, using the PICOS tool (Population, Intervention, Comparison, Outcome and Study type), associated with an assessment of the risk of bias and an evaluation of the results of the articles selected [20].

Only the first affiliation of the first author was considered in the geographical analysis of publications.

## Results

### Data collection

**Fig. 1:**
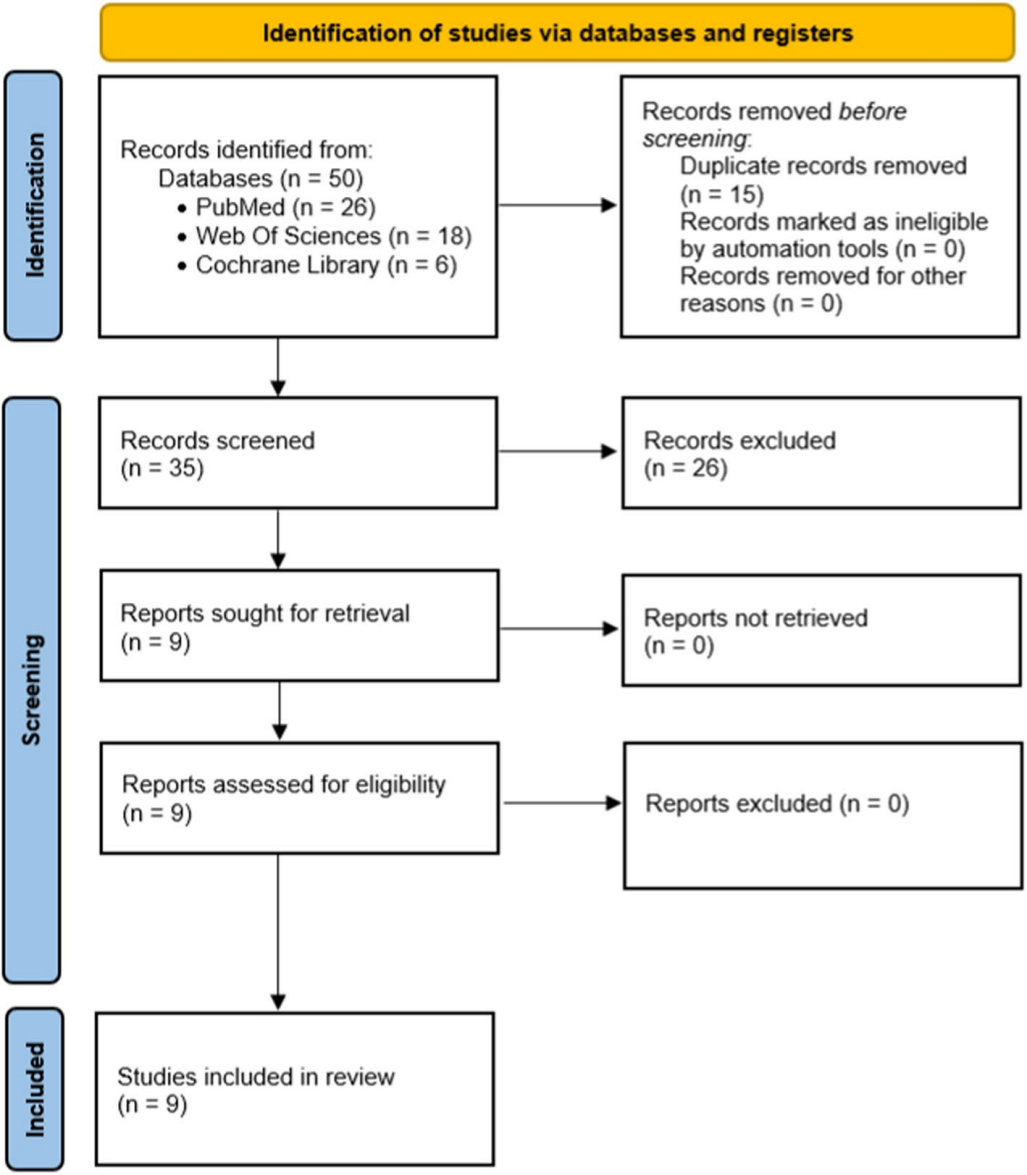
PRISMA flow diagram of the search of studies on evaluation of mandibular bone density and/or bone volume with imaging examination in adult patients

Thus, out of a total of 50 articles retained initially from the 3 databases, 15 duplicates were eliminated. 26 articles were excluded on the basis of their titles and abstracts. Nine articles were retained after reading the full texts and a total of nine articles corresponding to the inclusion criteria were selected and studied in this work (Fig. 1).

### Data extraction and analysis [21–29]

The study search strategy is shown in a flow chart (Fig. 1). Nine articles that met the inclusion criteria were selected. The characteristics for each data category were extracted into Table 2 (PICOS analysis, bruxism diagnosis and principal results). Methodological quality assessment of these nine studies is proposed in Table 3.

**Table 2:** Extraction of data from the selected articles

Studies selected	PICOS	Bruxism diagnosis	Principal results
Padmaja Satheeswarakumar, 2018, Assessment of Mandibular Surface Area Changes in Bruxers Versus Controls on Panoramic Radiographic Images: A Case Control Study [21]	(P) 40 adults aged 20–30 years diagnosed as bruxers or non-bruxers (I) Panoramic radiographs analysed with ImageJ® (C) Comparison of the surfaces of the mandible, the coronoid process and the condylar process between a group of bruxer patients and a control group of non-bruxer patients (O) To evaluate a change in bone surface area in the mandible, coronoid process and condylar process in bruxer patients compared to non-bruxer patients (S) Monocentric comparative observational study	Questionnaire and confirmation by the wearing of a Bruxchecker®	Statistically significant difference between bruxer and non-bruxer patients, in favour of bruxer patients: - Significant reduction in the surface area of the condylar processes, *p* < 0.05 - Significant reduction in the surface area of the right coronoid process, *p* < 0.05 - Significant increase in the surface area of the left coronoid process, *p* < 0.05 No statistically significant difference in mandibular area
Türp, 2021, Bone apposition at the mandibular angles as a radiological sign of bruxism: a retrospective study [22]	(P) 100 adults aged 21–83 years diagnosed as bruxers, 100 adolescents aged 12–18 years (control group) (I) Panoramic radiographs (C) Comparison of the presence or absence of directional change and macroscopically visible bone apposition at the mandibular angle (O) To evaluate the prevalence of macroscopically visible alterations at the mandibular angle and to detect different morphological characteristics of the gonial angles in bruxer patients compared to non-bruxer patients (S) Retrospective observational study (case–control)	Clinical examination	Statistically significant difference between the bruxer patients and the control group in favour of the bruxer patients: - The od ratio for unilateral apposition was 288 *p* < 0.0001 - The od ratio for bilateral apposition was 363 *p* < 0.0001
Isman, 2021, Evaluation of jaw bone density and morphology in bruxers using panoramic radiography [23]	(P) 120 adults aged 24–52 years diagnosed as bruxers or non-bruxers (I) Panoramic radiographs analysed with ImageJ® (C) Comparison of different mandibular cortical bone thicknesses and gonial shape changes between a group of bruxer patients and a control group of non-bruxer patients (O) To evaluate the effects of sleep bruxism on jaw bone density, mineralization and morphology in bruxer patients compared to non-bruxer patients (S) Monocentric comparative observational study	Self-administered questionnaire	- A significant association was observed between the shape of the inferior mandibular cortex and bruxer status (*p* < 0.012) - The width of the cortical bone below the mental foramen was significantly greater in bruxers (*p* < 0.006) - Presence of bony exostoses was associated with bruxer status (*p* < 0.001) -Antegonial notch depth was greater in bruxers than in non-bruxers (*p* < 0.001) - Cortical thickness at the gonial angle was significantly greater in bruxers (*p* < 0.001) - Panoramic mandibular index was not different in bruxers and non-bruxers (*p* > 0.05) - Thickness of the lower mandibular cortex opposite the anterior border of the ramus was not associated with bruxism status (*p* > 0.4)
Eninanc, 2021, Evaluation of the effect of bruxism on mandibular cortical bone using radiomorphometric indices on panoramic radiographs [24]	(P) 252 adults aged 18 to 45 years diagnosed as bruxers or non-bruxers (I) Panoramic radiographs, analysis software not specified (C) Comparison of 3 radiographic indices to assess mandibular cortical thickness between a group of bruxers and a control group of non-bruxers (O) To evaluate radiographic changes occurring in the mandibles of bruxers following exposure to prolonged strong occlusal forces (S) Monocentric comparative observational study	Self-administered questionnaire (at least 2 criteria of Pintado et al*.* [30]) Clinical examination (all criteria of Rompre et al*.* [31]) Patients examined by a single maxillofacial radiologist	- Mean MI values were significantly higher in bruxers than in the control group (*p* = 0.007) - no significant difference in MCI between bruxers and non-bruxers *p* > 0.05 - the difference between the groups in terms of mean PMI values was not significant (*p* > 0.05)
Gulec, 2021, Evaluation of the mandibular trabecular bone in patients with bruxism using fractal analysis [25]	(P) 212 adults aged 21–40 years diagnosed as bruxers or non-bruxers (I) Panoramic radiographs analysed with ImageJ® (C) Fractal analysis comparison of grey values of trabecular bone at the condyle, gonial angle and alveolar bone between a group of bruxer patients and a non-bruxer control group (O) To evaluate the effect of bruxism on the fractal dimension (density) of mandibular trabecular bone, and to evaluate the effectiveness of fractal analysis as a diagnostic test for bruxism (S) Monocentric comparative observational study	Self-administered questionnaire and clinical examination Examined by one person	- Right condyle measurements significantly smaller in bruxers *p* < 0.05 - No statistically significant difference found for the other parameters studied
Eninanc, 2021, Investigation of mandibular fractal dimension on digital panoramic radiographs in bruxist individuals [26]	(P) 252 adults aged 18–45 years diagnosed as bruxers or non-bruxers (I) Panoramic radiographs analysed with ImageJ® (C) To compare the internal trabecular structure of different jaw sites by measuring the fractal dimension on panoramic radiographs acquired with automatic exposure dosing between a group of bruxer patients and a non-bruxer control group (O) To evaluate changes in mandibular trabecular bone structure in bruxism using fractal analysis on digital panoramic radiographs obtained with automatic dosing (S) Monocentric comparative observational study	Self-administered questionnaire (at least 2 criteria of *Pintado *et al*.* [30]) Clinical examination (all criteria of *Rompre *et al*.* [31]) Patients examined by a single maxillofacial radiologist	- Mean fractal dimension values in bilateral gonial regions of bruxers were significantly lower than those of controls (*p* = 0.049) - Differences in fractal dimension values between groups were not significant in condylar and dental regions (*p* > 0.05)
Yilmaz, 2022, A new perspective for radiologic findings of bruxism on dental panoramic radiography [27]	(P) 209 adults diagnosed as bruxers or non-bruxers (I) Panoramic radiographs (C) Comparison of bone apposition at the mandible angle with a grade classification and Mandibular Cortical Index between a group of bruxer patients and a non-bruxer control group (O) To evaluate whether there is a relationship between the appositional classification in the mandible angle region and the mandibular cortical index (S) Retrospective cross-sectional study	Self-administered questionnaire (at least 2 criteria of Pintado et al*.* [30]) and clinical examination	- A statistically significant difference was found between MCI with bruxer and non-bruxer groups (*p* < 0.001) - There is no statistically significant difference between MCI and grades (*p* = 0.063) in bruxers
Serafim, 2022, Impact of bruxism on craniomandibular morphology: A cone-beam computed tomographic study [28]	(P) 70 adults aged 18 to 44 years diagnosed as bruxers or non-bruxers (I) Cone Beam Computed Tomography analysed with ITK-SNAP 3.4.0 software (C) To evaluate the angle between the mandibular ramus and body and condylar volume,comparing the right and left sides (O) To evaluate morphological changes in the mandible concerning in bruxers and non-bruxers patients (S) Retrospective cross-sectional study	Clinical examination	- Significant difference was observed between bruxer and non-bruxers: right and left gonial angles were smaller in bruxers - No significant difference was observed between the groups with or without bruxism in relation to the right condylar volume (*p* = 0.956) and the left condylar volume (*p* = 0.740)
Casazza, 2023 Evaluation of mandibular bone density in bruxers: the value of panoramic radiographs [29]	(P) 84 adults aged 19 to 84 years diagnosed as bruxers or non-bruxers (I) Panoramic radiographs analysed with ImageJ® (C) Comparison of the ratio of cancellous bone to cortical bone (in grey values) and presence or absence of bony exostoses at the mandibular angle between a group of bruxers and a control group of non-bruxers (O) To evaluate a difference in bone density at the level of the 1st mandibular premolar and the presence of bony exostoses at the mandibular angle between bruxers and non-bruxers (S) Retrospective cross-sectional study	Self-administered questionnaire Clinical examination by calibrated practitioners	Statistically significant difference between bruxer and non-bruxer patients, in favour of bruxer patients: - Higher cancellous to cortical bone ratio, *p* < 0.01 - Greater cancellous bone density, *p* < 0.01 - Higher number of bony exostoses at the mandibular angle, *p* < 0.01

**Table 3:** Methodological quality assessment

Studies	Calculation of the required number subjects	Bruxism diagnosis [1]	Assessment method of outcome	Several evaluators	Blinding assessment of outcome	Patients selection bias
Padmaja Satheeswarakumar et al*.*, 2018 [21]	No	Probable	Moderate risk of bias	Yes	Not specified	No
Türp et al., 2021 [22]	No	Probable	Moderate risk of bias	Yes	Not specified	Yes
Isman, 2021 [23]	Yes	Possible	Moderate risk of bias	Yes	Yes	No
Eninanc et al*.*, 2021 [24]	Yes	Probable	Moderate risk of bias	No	No	No
Eninanc et al*.*, 2021 [26]	Yes	Probable	Moderate risk of bias	No	No	No
Gulec et al*.*, 2021 [25]	Yes	Probable	Moderate risk of bias	Yes	Yes	No
Yilmaz et al*.,* 2022 [27]	Yes	Probable	Moderate risk of bias	Yes	Yes	No
Serafim et al*.*, 2022 [28]	Yes	Probable	Low risk of bias	No	Yes	No
Casazza et al*.*, 2023 [29]	Yes	Probable	Moderate risk of bias	Yes	Yes	No

The papers included in the final step of the review covered a wide geographical area, including the Middle East (Turkey, *n* = 5 studies), South America (Brazil, *n* = 1), Asia (India, *n* = 1), and Europe (France, *n* = 1, Switzerland, *n* = 1). All studies were approved by the ethics committee of their respective institutions or hospitals. The strategies adopted to study bone characteristics were different, with different types of radiological examinations, so a direct comparison of bone density and bone volume could not be performed. The details of these different results are recorded in Tables 2 and 3.

### Synthesis of results

Using the PICOS tool, it was determined that five studies described monocentric comparative observational studies, while four were retrospective studies. The nine studies selected evaluated at least one mandibular bone parameter in bruxers and in a control group of non-bruxers. The total sample observed comprised 1,187 adult patients. Two studies [24, 26] had the same registration number for their controlled clinical trials, with the same number of patients. One of these studies investigated mandibular cortical bone [24], and the other mandibular cancellous bone [26], and these studies differed in the parameters and techniques used to assess the effects of bruxism on the different areas of interest. Thus, the total number of patients included in these studies was counted only once in calculating the overall number of patients studied in this scoping review.

The method(s) used for the diagnosis of bruxism differed between studies (medical interrogation [21], self-report assessment [23–27, 29], clinical examination [22, 24–29] use of the Bruxchecker© [21]). One study did not provide details about the clinical examination used to diagnose bruxism [28]. A more detailed characterization of bruxism episodes incorporating the patient's state of consciousness (awake or asleep), the presence of dental manifestations (clenching, grinding, tapping, or jiggling), or periods of bruxism activity (active and/or past bruxism episodes at the date of the patient's visit) was not reported.

All the selected studies except one [28] used panoramic radiographs, which are two-dimensional imaging examinations, to investigate the characteristics of the mandibular bone. Five of them [21, 23, 25, 26, 29] used the same imaging analysis software: ImageJ®. Three studies [22, 24, 27] did not use analysis software. However, the parameters studied differed greatly between studies: 1 out of 9 studies evaluated the surface area of certain areas of the mandible [21, 22], 3 studies evaluated presence of exostoses in the mandibular angle [22, 27, 29], 3 studies evaluated measurements associated to bone morphological characteristics (e.g. bone thickness or width of mandible, condylar volume) [23, 24, 28], 3 studies evaluated the different grey values in the selected regions of interest, using various analytical methods (fractal dimensions, grey value averages, grey value ratios) [25, 26, 29].

The 9 selected studies found a significant difference between bruxers and non-bruxers for at least one parameter studied.

## Discussion

This scoping review, which focuses on the difference in density or volume of the mandibular bone in adult patients diagnosed as bruxers, identified nine scientific articles published in the international literature.

The general area of interest of these studies was the evaluation of a potential modification of mandibular bone in patients diagnosed as bruxers compared to a control group of non-bruxers. Indeed, the forces generated by bruxism can significantly exceed the amplitude of the maximum voluntary occlusal force during wakefulness [11]. According to Wolff's law, following a stress, the bone adapts, bone remodelling in bruxers should therefore be observed [15]. The best research approach to address this problem is in vivo research, in humans: the optimal method for studying these bone variations is therefore medical imaging. Indeed, medical imaging is the only non-invasive and painless way to observe changes in bone structure. Eight studies selected used panoramic radiographs. This introduced a reading bias with the risk of superimposition of structures, since an initially three-dimensional structure is studied in two dimensions. However, this information could be collected following a control examination, since the justification for using X-rays for the sole purpose of screening for bruxism is not currently established. It would be beneficial in the long term to collect more precise three-dimensional data through CT scans and Cone Beam Computed Tomography (CBCT), in order to justify the use of CBCT for the diagnosis of bruxism. In the literature review, only one study used CBCT made for orthodontic patients [28]. Thus, this path, so far unexploited, is beginning to arouse the interest of research teams. Investigations in this field should therefore be pursued.

Furthermore, it appears that despite the 2018 consensus on the definition of bruxism, it remains difficult to diagnose definitively, with poorly established diagnostic criteria [32]. Indeed, there is no precise and universally recognized diagnostic method, although several approaches are used (self-administered questionnaire, clinical examination, polysomnography and electromyography). Each has its advantages and disadvantages [4]. Based on the classification system proposed by Lobbezoo et al. [4], most of the selected studies presented a "probable" diagnosis of bruxism, each with a different approach to history-taking and very disparate clinical criteria. In their clinical review, the study by Gulec et al*.* used only one criterion: tooth wear [21]. It is however recognized that this criterion is not exclusively related to bruxism but can originate from other wear processes such as abrasion and erosion [33], harmful lifestyle habits, physiological aging of the tooth [4]. Moreover, in order to limit diagnostic error, only Casazza et al*.* confirmed the reproducibility of their clinical examination via the calibration of several practitioners. A similar disparity appears in the wording of the patient self-administered questionnaires used in some studies for the diagnosis of bruxism. All these different factors therefore generate a bias in the selection of patients and their allocation to their respective case or control group, which seems likely to compromise the validity of the results presented. However, these difficulties could soon be solved thanks to the research and synthesis work carried out by an international group of recognized specialists with the aim of proposing a tool allowing reliable and feasible way for the evaluation of bruxism. In fact, in a very recent article published after the studies selected in this scoping review, Manfredini et al*.* present the “Standardised Tool for the Assessment of Bruxism”, or STAB [34]. STAB is divided into two axes. The first axis includes the self-reported information, clinical and instrumental assessment on bruxism status and its potential consequences. The second axis includes the self-reported information on factors that may have an etiological or comorbid role for bruxism. This tool has yet to be tested in daily practice and in research but should constitute a common diagnostic basis on which research teams working on bruxism could rely on. In addition, four selected studies were retrospective studies, which may have contained some additional bias. Indeed, these studies were dependent on the maintenance of medical records and the collection of radiological examinations, but in most cases did not allow the implementation of a clinical examination of the patient or a standardized questionnaire. Thus, the analysis of medical imaging at a given time did not allow us to judge whether the changes observed were due to primary morphology, physiological aging, or actually to the patient's bruxism.

Of the nine selected studies, all found a significant difference between bruxers and non-bruxers, examining different regions of the mandible, and using different medical imaging analysis techniques, which may have impacted the results presented. Of these nine studies, six of them [17, 19–22] presented contrasting results depending on the parameters studied: some showed a statistically significant difference between bruxers and non-bruxers, others did not. These differences in results could be found for the same factor studied between the right and left sides of the selected panoramic radiographs, which raised questions about the reliability of certain established correlations.

In the mandibular condyle region, Padmaja Satheeswarakumar et al*.*,Gulec et al*.* with panoramic radographs and Serafim *and al.* [28] with CBCT observed a significant reduction in the surface area or volume of the condylar processes in bruxer patients [21, 25, 28]. At first glance, this decrease in volume seems counter-intuitive, as bruxism is usually associated with sturdy condyles. A possible explanation is that the forces generated during bruxism episodes, through their frequency and intensity, could cause degeneration of the bone tissue in this area, exceeding the capacity of the mandibular condyles to adapt to the loads applied to them. On the other hand, Eninanç et al*.* [26] did not find any differences in mandibular condyles between bruxers and non-bruxers with their fractal dimension analysis, which would indicate that there is no change in trabecular bone structure in bruxism. In the coronoid process region, Padmaja Satheeswarakumar et al*.* observed coronoid process measurements in bruxers that were significantly smaller on the right but larger on the left. On the other hand, Cezairli et al*.* [35]*,* whose study was not included among the selected articles because it included a 15-year-old patient, found that the height and width of the left and right coronoid processes were significantly greater in bruxers than in non-bruxers. More studies are needed to support a possible difference, whether or not in favour of the bruxer group.

In the gonial angle region, the presence of greater number of exostoses could probably be associated with bruxer status as this represented a statistically significant observation in the study by Isman [23] and Casazza et al*.* [29]. Türp et al. [22] and Isman [23] observed greater thickness and density of gonial bone in bruxers, while Eninanc et al*.* found smaller values for gonial bone density and thickness in bruxers [26]. Serafim et al. [28] observed a decreased mandibular angle in CBCT in bruxers, which may be associated with the insertion of the masseter and medial pterygoid muscles [36, 37]. In the area of the cancellous bone of the mandibular premolars, Casazza et al. [29] found a significantly higher density in bruxers. This was also observed by Shokry et al. [38]. However, Eninanç et al*.* [26] did not find statistically significant differences between the bruxer and non-bruxer groups in the two areas of the mandibular body between the apical areas of the first molar and second premolar and between the first premolar and canine. The preferential use of the premolar region to obtain measurements is explained by the smaller presence of anatomical elements or superimpositions in this region on panoramic radiographs likely to induce biases in the measurements carried out, unlike other areas such as the mandibular symphysis or the molar region, where projections are found that are likely to impair the quality of the measurements carried out, and which thus cannot be used on panoramic radiographs. For this reason, the use of three-dimensional imaging would allow us to overcome these obstacles and to perform usable measurements in different mandibular bone locations. These results should be confirmed by other studies that will overcome this limitation and allow the results to be extended to other regions of interest in the mandible. Concerning mandibular cortical bone, several measurements in different studies have shown statistically significant differences between bruxer and non-bruxer patients. Indeed, Isman and Yilmaz et al. [23, 27] using the same index (MCI) showed a difference in mandibular cortical shape with directional changes. The mandibular cortical bone width was significantly different between the bruxers and non-bruxers in two studies [23, 24].

### Limitations

A scoping review was chosen by the authors to identify the types of evidence available in the field of research on the impact of bruxism on the mandibular bone and to analyse the knowledge gaps. The main limitation of this review lies in the search strategy. Indeed, it may have prevented the identification of all studies of interest because of limitations in database coverage and to the particularities of article indexing. Moreover, the articles from the grey literature were not taken into account: perhaps other studies could have been found by this means.

The possibility of comparing the results of these different studies is therefore limited because of their great methodological disparity and the small number of articles available. For this reason, it was not possible to carry out a meta-analysis on the basis of this review.

## Conclusion

Nine studies have been selected in this scoping review. They evaluated at least one mandibular bone parameter in bruxers and in a control group of non-bruxers patients. All of them found a significant difference between the two groups for at least one parameter studied. Bruxism seems to induce morphological and anatomical changes in the different regions of the mandibular bone (condyles, mandibular angle, mandible body). Interpretation of results is limited by the dearth of current studies on this subject. However, several avenues of research seem promising, for example, the greater number of bony exostoses at the mandibular angle, condylar morphology (smaller condyles), and the quality of cortical and cancellous bone, and these avenues deserve to be explored more fully. Such research would allow the integration of medical imaging, as an additional element to be considered, into the rationales established by the practitioner or researcher for the diagnosis of bruxism. Particular attention should be paid to three-dimensional medical imaging, such as CBCT (Cone Beam Computed Tomography) in order to avoid certain biases common to all the studies included in this literature review, in which only 2D radiological analysis (panoramic X-ray) was used. However, since CBCT is not a reference radiological procedure for the diagnosis of bruxism, its possible justification can only be established following the analysis of CBCT initially indicated for other pathologies or oral therapeutic procedures. Given the high prevalence of bruxism in the general population, a better knowledge of the variations in bone density in these patients would be of real value for their management in certain fields of dentistry such as dentofacial orthopaedics, periodontology or implantology. Prospective clinical study analysing CBCT has just been initiated and may provide results about bone density. Consequently, if the hypothesis is verified, CBCT could become a complementary radiological examination to aid in the diagnosis of bruxism.

## Data Availability

The datasets used and/or analysed during the current study are available from the corresponding author on reasonable request.
